# Long Term Prospective Assessment of Living Kidney Donors: Single Center Experience

**DOI:** 10.1155/2014/502414

**Published:** 2014-01-23

**Authors:** Ayman Maher Nagib, Ayman Fathi Refaie, Yasser Abdelmoniem Hendy, Magdy Abass Mohmed Elfawal, Ahmed Abdelrahman Shokeir, Mohamed Adel Bakr, Ahmed Hassan Neamattala, Ahmed Farouk Hamdy, Khaled Mohamed Mahmoud, Amani Mostafa Ismail, Mohamed Ahmed Ghoneim

**Affiliations:** ^1^Department of Nephrology, The Urology and Nephrology Center, Mansoura 35516, Egypt; ^2^Department of Nephrology, Zagazig University, Zagazig 44519, Egypt; ^3^Department of Radiology, Zagazig University, Zagazig 44519, Egypt; ^4^Department of Urology, The Urology and Nephrology Center, Mansoura 35516, Egypt; ^5^Department of Immunology, The Urology and Nephrology Center, Mansoura 35516, Egypt

## Abstract

Virtually, all studies reporting the outcomes of living kidney donation beyond the first year from donation were retrospective. In this prospective study, the outcome of 81 consecutive living kidney donors was thoroughly evaluated. Clinical, laboratory, and radiological assessments were carried out at predonation (basal), 3, 6, 12, and 24 months after donation. The mean age at time of donation was 37.8 ± 9.8 years and the majority was females (75.3%). The mean BMI increased significantly after donation (*P* < 0.04). The mean serum creatinine levels (mg/dl) were 0.75 ± 0.14, 1.01 ± 0.22, 0.99 ± 0.21, 0.98 ± 0.20, and 0.94 ± 0.20 (*P* < 0.0001). Likewise, the mean levels of measured creatinine clearance (mL/min) were 148.8 ± 35.7, 94.7 ± 26.6, 95.5 ± 24.6, 96.7 ± 20.2, and 101.6 ± 26.2 (*P* < 0.0001). The mean 24 hours urinary protein excretion (gm/dL) were 0.09 ± 0.03, 0.19 ± 0.18, 0.16 ± 0.09, 0.18 ± 0.25, and 0.17 ± 0.12 (*P* < 0.0001). There were significant increases in the means of the longitudinal and transverse diameters of the remaining kidney over time (*P* < 0.001). Out of 42 female donors, eleven female donors have got successful postdonation pregnancies. There were no reported surgical complications, either intra- or postoperative. Long-term follow-up is necessary for all living kidney donors through local institutional and world registries. This trial is registered with ClinicalTrials.gov NCT00813579.

## 1. Introduction 

Living donor kidney transplantation is the treatment of choice for patients with end-stage renal failure for several reasons. The transplant is performed when the donor and the recipient are in optimum medical condition and at a time that is convenient for them and for their families. Moreover, recipients of living donor kidney grafts enjoy greater long-term graft survival and a better quality of life than do recipients of cadaveric kidney grafts [1]. It reduces the number of patients on the wait list for a cadaveric kidney and therefore increases the likelihood that patients with no potential living donor can undergo transplantation. This is particularly important because the gap between the number of cadaveric donors and the number of patients on the waiting list is increasing due to significant reduction in traffic accidents as well as the promotion of more healthy lifestyles with emphasis on exercise and improved dietary habits and the subsequent reduction in the incidence of stroke [2]. An increase in the number of living donors (including living-unrelated donors) may ameliorate this trend [3].

Almost all studies that report medical outcomes of living kidney donors more than a year from donation are retrospective. Although practical for evaluation of long-term outcomes, retrospective studies are vulnerable to certain methodological pitfalls and biases that may limit their interpretability. Most important is the potential for selection bias, which may alter findings if there is a difference between included and nonincluded donors either as a result of non-participation or because the study investigators are unable to locate the subject. In studies that follow living kidney donors, donors in good health may be more likely to participate because of greater survival or greater ability to meet the requirements of participation [4].

The aim of this study is to evaluate the impact and safety of kidney donation on living kidney donors in a prospective fashion.

## 2. Materials and Methods 

Donors included in this study were male or female and donors were of a minimum of 21 years and a maximum of 65 years of age. Donor has been fully informed and has given written informed consent.

While criteria for exclusions were donor has hypertension or diabetes mellitus, donor is pregnancy or breastfeeding, donor is known to be HCV, HBV, and HIV positive, donor has significant liver disease, defined as having during the past 28 days continuously elevated AST and/or ALT levels 3 times greater than the upper value of the normal range of the investigational site, donor with malignancy or history of malignancy, or active systemic or localized major infection, donor is participating or has participated in another clinical trial, evidence of infiltrate, cavitation or consolidation on X ray obtained during the screening/baseline evaluation, subjects with a screening/baseline hemoglobin < 11 gm/dL, total white blood cell count ≤ 2,000/mm^3^, and platelet count ≤ 100000/mm^3^, fasting triglycerides ≥ 400 mg/dL, or fasting total cholesterol ≥ 300 mg/dL.

In this prospective study, the outcome of 81 consecutive living kidney donors, who donated their kidneys between December 2007 and November 2008 in our center, was thoroughly evaluated. Clinical, laboratory, and radiological assessments were carried out at pre-donation (basal), 3, 6, 12, and 24 months after donation Qualitative data were displayed in cross tabulation and quantitative data were described in terms of arithmetic mean ± SD. Bivariate techniques were used for initial evaluation of contrasts. A *P* value of ≤ 0.05 was considered significant.

## 3. Results 

The mean age at time of donation was 37.8 ± 9.8 years (range: 22–64 years) and the majority were females (61, 75.3%). They were 45 parents (37 mothers), 28 siblings (19 sisters), and 8 emotionally related (5 wives). Right nephrectomy was carried out in 40 donors. The mean BMI (kg/m^2^) increased significantly after donation. There were no significant differences between the means of systolic and diastolic blood pressures while 4 donors out of the 81 donors developed mild hypertension which was easily controlled on one antihypertensive drug (Table 1).

The mean serum creatinine levels increased significantly in the first three months after donation and afterwards it decreased significantly till the end of the observation period.

Likewise, the mean levels of both measured and estimated creatinine clearance either by Cockcroft and Gault equation or by MDRD decreased significantly in the first three months after donation afterwards it increased significantly till the end of the study. The urinary protein excretion estimated either by 24 hours urine collection or protein creatinine ratio increased significantly in the first three months after donation and afterwards it stabilized over the remaining period of the study (Table 2).

After donation, biochemical changes over the first 24 months (Table 3) were regularly monitored. There was significant increase in the levels of the serum uric acid three months after donation, which was stabilized afterwards.

The blood sugar, serum calcium, phosphorus, liver enzymes ALT, AST, total protein, serum albumin, and bilirubin remained within normal levels throughout the observational period.

There were modest increases in the levels of the serum triglycerides and cholesterol after donation.

There was a significant increase in the means of the longitudinal diameter (cm) of the remaining kidney at basal, 3, 6, 12, and 24 months 10.93 ± 0.88, 11.53 ± 0.9, 11.55 ± 0.79, 11.67 ± 0.73, 11.79 ± 0.76, respectively (*P* < 0.001). Also, transverse diameters (cm) of the left kidney increased significantly over time 4.21 ± 0.59, 4.51 ± 0.62, 4.54 ± 0.64, 4.61 ± 0.58, and 4.84 ± 0.50, respectively (*P* < 0.001).

Out of 42 female donors, eleven have got successful post donation pregnancies.

There were no reported surgical complications either intraoperative or postoperative.

## 4. Discussion

Although practical for evaluation of long-term outcomes, retrospective studies are vulnerable to certain methodological pitfalls and biases that may limit their interpretability [4]. So, our study was designed in prospective way. The policy in the Urology and Nephrology Center, Mansura University, Egypt, is to perform kidney transplantation from related donors. Strict ethical policies and rules of the international community were strictly followed to ensure the protection and safety of living donors and appropriate recognition for their heroic act while combating transplant tourism, organ trafficking, and transplant commercialism [5].

In this study, most of the donors were female (75.3%), and this in accordance with that reported previously by Biller-Andorno, 2002 [6], being approximately 65% of live kidney donors, were women and approximately 65% of recipients were men. This perhaps is reflecting a psychological submission or discrimination of women in many countries, including western nations. In our study, most of the donors were mothers, sisters, and wives and this could explain the predominance of female donors. Furthermore, since it is reported that the incidence of end stage renal disease is more frequent among males this may partly explain why there are more wives than husbands who donate in the case of transplants between spouses [6].

All our donors except four enjoyed normal blood pressure throughout the observational period and the hypertension was mild and easily controlled with single antihypertensive agent.

The majority of the studies that assess hypertension in living kidney donors were retrospective and did not include control groups that were assembled and followed along with donor controlled studies in which the average follow-up was at least [7, 8].

Years after donation (range: 6 to 13 years), blood pressure was 5 mm Hg higher in donors than in control participants [9]. It is worth to mention that the age of these donors was above fifty and developed progressive increase in body weight after donation which makes them vulnerable to develop hypertension.

In this study, the mean serum creatinine levels increased significantly in the first three months after donation. Afterwards, there was a significant improvement till the end of the observation period. Likewise, the mean levels of measured and estimated creatinine clearance follow the same pattern of improvement. Samhan and Omar, 1999 [10], studied the early changes in kidney function following live kidney donation. They reported an immediate and significant increase in effective renal plasma flow (ERPF) of the remaining kidney. The median value increased from 50.4% to 90% of the preoperative total ERPF at 3 months after donation.

The median levels of serum creatinine concentrations increased by 30% in comparison with the prenephrectomy level likewise the median values of creatinine clearance at 3 months after the donor nephrectomy dropped down to 76% of the prenephrectomy value. This reduction in creatinine clearance corresponds with the persistent elevation of serum creatinine concentration.

Garg et al., 2006 [11], reviewed studies which reported GFR in categories, 12% of donors developed a GFR between 30 and 59 mL/min (range: 0–28%), and 0.2% a GFR less than 30 mL/min (range: 0–2.2%). An initial decrease in GFR after donation was not accompanied by accelerated losses over that anticipated with normal aging. An initial decrease in GFR is not followed by accelerated losses over a subsequent 15 years. Future studies will provide better estimates and identify those donors at least risk of long-term morbidity.

In our study, there was a compensatory hypertrophy of the remaining kidney evidenced by increase in the both longitudinal and transverse diameters of the remaining kidney overtime during the observational period. In accordance to our results, Bohlouli et al., 2010 [12], reported that remnant kidney length, anterior-posterior diameter, and cortical thickness were significantly increased during postnephrectomy follow-up. This compensatory hypertrophy could explain the improvement of kidney function which starts to occur at the end of the study. Urinary protein excretion was increased in the first three months after donation. Afterwards, it stabilized over the remaining period of the study.

Previous reports showed that there were no significant changes. In urinary protein in the first three months after donation, these results in contrary to our results and may due to progressive and significant increase in the BMI in among our donors.

It is reported that, after nephrectomy, there is a compensatory hemodynamic change; also there has been a concern that kidney donors (who undergo a 50% reduction in renal mass with donation) might have compensatory glomerular hyperfiltration and hypertrophy.

Garg et al., 2006 [11], reviewed forty-eight studies from 27 countries followed a total of 5048 donors.

An average of 7 years after donation (range: 1–25 years), the average 24 hours urine protein was 154 mg/day so kidney donation results in small increases in urinary protein. Our results were similar to those which were reported previously. All the donors except one enjoyed within normal blood glucose levels. The donor who developed postdonation hyperglycemia was a 48-year-old mother, who showed a predonation normal glucose tolerance curve and developed postdonation progressive increase in body weight, beside her positive family history for diabetes mellitus. These two risk factors could explain her new onset diabetes mellitus post donation. Boudville and Isbel, 2010 [13], reported that this incidence of postdonation DM ranging from 1.7 : 7.4% with a follow-up of more than 20 years in some studies.

Ibrahima and associates reported that kidney donors, similar to the general population, are at risk for development of type 2 diabetes mellitus (T2DM). The course of donors who develop T2DM has not been studied. They surveyed 3777 kidney donors regarding the development of T2DM. Of the 2954 who responded, 154 developed T2DM 17.7 ± 9.0 years after donation. The multivariable risk of development of T2DM was associated with type 1 DM in the recipient, male gender, and body mass index > 30 kg/m^2^ at time of donation. These preliminary and short-term data demonstrate that factors associated with T2DM in kidney donors are similar to those in the general population and donors screened carefully at the time of donation do not appear to have an acceleration diabetic kidney disease [14].

Significant increases in the levels of serum uric acid in the first three months after donation were reported in our donors, which could be explained by the mild transient impaired renal function in the early postdonation period.

In the current study, the lipid profile changed variably through the first three months after donation. There was a significant increase in the mean levels of serum triglycerides which was stabilized afterwards. On the other hand, there were no significant differences in the mean levels of serum total cholesterol, LDL, or HDL in the first three months, but their levels showed significant increase at 3, 6, 12, and 24 months. According to Tavakol et al., 2009 [15], obese donors were more likely to have abnormal HDL levels after donation. This could explain the increase of serum lipids among our donors who developed postdonation significant increase in body mass index.

It is worth mentioning that 11 female donors have got successful postdonation pregnancies. In accordance, Ibrahim et al., 2009 [16], reported that pregnancy in kidney donors has generally been viewed to be favorable and determined fetal and maternal outcomes in a large cohort of kidney donors. In accordance to our study, Ibrahim and their colleagues, 2009 [17], stated that a total of 2102 women have donated a kidney at their institution: 1589 donors responded to pregnancy surveys, 1085 reported pregnancies, and 504 reported none. In this large survey of previous living donors in a single center, fetal and maternal outcomes and pregnancy outcomes after kidney donation were similar to those reported in the general population.

## 5. Conclusion 

Obese potential live kidney donors should be advised to maintained ideal body weight in order to avoid proteinuria, hypertension, and diabetes mellitus. Proteinuria increases with marginal significance but appears to be of no clinical consequence. Despite the reduction in GFR in the early post donation period, afterwards it increased to normal values.

Although living kidney donation is a safe procedure which carries a minimal risk in comparison with the heroic act and the major benefits for the transplant recipients, the proper selection and the pretransplant assessment of the potential kidney donors together with postdonation meticulous and regular (lifelong) follow-up is strongly recommended.

Future controlled, prospective studies with long periods of follow-up will better delineate safety and identify donors at lowest risk for long-term morbidity. A world registry of living donors is necessary to evaluate the real magnitude of the long-term risk to living donors.

## Figures and Tables

**Table 1:** Clinical data at different intervals.

Variables	Basal	At 3 months	At 6 months	At 12 months	At 24 months	*P* value
BMI (Kg/m^2^)	29.72 ± 5.37	30.13 ± 5.72	30.44 ± 5.82	30.85 ± 5.82	30.99 ± 6.13	0.004
Blood pressure (mmHg)	120.8 ± 6.49	124.1 ± 11.7	121.9 ± 12.5	120.9 ± 11.6	118.5 ± 15.9	0.22
Systolic Diastolic	79.2 ± 4.9	80.5 ± 7.1	79.2 ± 8.27	79.1 ± 8.5	77.1 ± 9.6	0.117

**Table 2:** Changes of the renal function tests in the first 24 months after donation.

Variables	Basal	At 3 months	At 6 months	At 12 months	At 24 months	*P* value
Serum creatinine (mg/dL)	0.75 ± 0.14	1.01 ± 0.22	0.99 ± 0.21	0.98 ± 0.20	0.94 ± 0.20	<0.001
Calculated creatinine clearance (mL/min)	148.8 ± 35.7	94.68 ± 26.6	95.49 ± 24.6	96.69 ± 20.2	101.6 ± 26.2	<0.001
Cockcroft and Gault (mL/min)	132.8 ± 36.2	101.5 ± 25.6	105.2 ± 27.3	106.7 ± 25.8	111.5 ± 29.6	<0.001
MDRD (mL/min)	107.2 ± 19.3	79.4 ± 20.5	80.3 ± 16.6	81.5 ± 17.7	84.4 ± 17.5	<0.001
24 hours urine protein (gm/day)	0.09 ± 0.03	0.19 ± 0.08	0.16 ± 0.09	0.18 ± 0.05	0.17 ± 0.02	<0.001
Protein creatinine ratio	0.09 ± 0.04	0.16 ± 0.04	0.14 ± 0.03	0.15 ± 0.03	0.17 ± 0.09	<0.001

**Table 3:** Changes of the biochemical values in the first 24 months after donation.

Variable	Basal	At 3 months	At 6 months	At 12 months	At 24 months	*P* value
FBS (mg/dL)	90.01 ± 9.73	91.09 ± 11.35	88.84 ± 14.72	84.05 ± 15.43	79.49 ± 22.9	0.41
PPBS (mg/dL)	105.46 ± 14.6	95.33 ± 10.6	102.3 ± 16.4	98 ± 9.1	103.7 ± 20.1	0.52
Total cholesterol (mg/dL)	179.3 ± 33.57	182.8 ± 31.6	185.6 ± 34.34	189.9 ± 40.34	192.4 ± 39.4	0.018
HDL (mg/dL)	46.62 ± 9.99	46.59 ± 10.81	44.95 ± 10.54	43.53 ± 9.95	45.47 ± 10.93	0.003
LDL (mg/dL)	116.89 ± 43.4	111.58 ± 27.64	115.93 ± 28.82	118.7 ± 31.38	119.93 ± 29.61	0.052
Triglycerides (mg/dL)	96.99 ± 50.23	115.8 ± 64.2	128.7 ± 85.5	121.02 ± 56.8	121.02 ± 56.8	0.003
Uric acid	4.5 ± 1.03	5.2 ± 1.1	5.27 ± 1.19	5.18 ± 1.16	5.37 ± 1.14	<0.001

## References

[1] Cecka JM (2002). The UNOS renal transplant registry. *Clinical Transplants*.

[2] Rothenberg L, Danovitch GM (2001). Ethical and legal issues in kidney transplantation. *Handbook of Kidney Transplantation*.

[3] Davis CL (2004). Evaluation of the living kidney donor: current perspectives. *American Journal of Kidney Diseases*.

[4] Ommen ES, Winston JA, Murphy B (2006). Medical risks in living kidney donors: absence of proof is not proof of absence. *Clinical Journal of the American Society of Nephrology*.

[5] Delmonico FL (2009). The implications of Istanbul Declaration on organ trafficking and transplant tourism. *Current Opinion in Organ Transplantation*.

[6] Biller-Andorno N (2002). Gender imbalance in living organ donation. *Medicine, Health Care, and Philosophy*.

[7] Saran R, Marshall SM, Madsen R, Keavey P, Tapson JS (1997). Long-term follow-up of kidney donors: a longitudinal study. *Nephrology Dialysis Transplantation*.

[8] Textor SC, Taler SJ, Driscoll N (2004). Blood pressure and renal function after kidney donation from hypertensive living donors. *Transplantation*.

[9] Boudville N, Prasad GVR, Knoll G (2006). Meta-analysis: risk for hypertension in living kidney donors. *Annals of Internal Medicine*.

[10] Samhan M, Omar AM, Al-Sae’ed T, Al-Mousawi M (1999). Early changes in kidney function following living donor nephrectomy. *Transplantation Proceedings*.

[11] Garg AX, Muirhead N, Knoll G (2006). Proteinuria and reduced kidney function in living kidney donors: a systematic review, meta-analysis, and meta-regression. *Kidney International*.

[12] Bohlouli A, Tarzamni MK, Zomorodi A, Abdollahifard S, Hashemi B, Nezami N (2010). Remnant kidney function and size in living unrelated kidney donors after nephrectomy. *Saudi Journal of Kidney Diseases and Transplantation*.

[13] Boudville N, Isbel N (2010). Donors at risk: impaired glucose tolerance. *Nephrology*.

[14] Ibrahima H, Kuklaa A, Cordnerb G, Baileyb R, Gillinghamb K, Matasb AJ (2010). Diabetes after kidney donation. *The American Journal of Transplantation*.

[15] Tavakol MM, Vincenti FG, Assadi H (2009). Long-term renal function and cardiovascular disease risk in obese kidney donors. *Clinical Journal of the American Society of Nephrology*.

[16] Ibrahim HN, Foley R, Tan L (2009). Long-term consequences of kidney donation. *The New England Journal of Medicine*.

[17] Ibrahim HN, Akkina SK, Leister E (2009). Pregnancy outcomes after kidney donation. *The American Journal of Transplantation*.

